# Effects of GLP-1 Receptor Agonists vs Metformin in Polycystic Ovary Syndrome: A Systematic Review and Meta-Analysis

**DOI:** 10.7759/cureus.103927

**Published:** 2026-02-19

**Authors:** Khuloud Almubaddil, Manal Alotaibi, Lena Bin Mutreb, Fouz Alanazi, Msaad Altulihee

**Affiliations:** 1 Family Medicine Department, King Saud University Medical City, Riyadh, SAU; 2 Family Medicine Department, King Fahad Medical City, Riyadh, SAU

**Keywords:** glp-1 receptor agonists, homa-ir, hyperandrogenism, insulin resistance, meta-analysis, metformin, pcos, reproductive health, systematic review

## Abstract

Polycystic ovarian syndrome (PCOS) is a common endocrine disorder associated with significant metabolic and reproductive dysfunction. Although metformin has long been a cornerstone of PCOS management, glucagon-like peptide-1 (GLP-1) receptor agonists (RAs) have emerged as promising alternatives, particularly for improving metabolic outcomes. This systematic review and meta-analysis aimed to evaluate the effects of GLP-1 RAs compared with metformin on metabolic, hormonal, and reproductive parameters in women with PCOS. Following the Preferred Reporting Items for Systematic Reviews and Meta-Analyses (PRISMA) guidelines, randomized controlled trials (RCTs) comparing GLP-1 RAs with metformin in women diagnosed with PCOS were systematically identified and analyzed. Four eligible RCTs comprising a total of 218 participants were included. Statistical analyses were conducted using a fixed-effects model. Compared with metformin, GLP-1 RAs were associated with significant reductions in serum testosterone (standardized mean difference (SMD) = −0.327, p = 0.036), dehydroepiandrosterone sulfate (DHEA-S) (SMD = −0.528, p = 0.048), androstenedione (SMD = −0.523, p = 0.002), and insulin resistance as assessed by the homeostasis model assessment of insulin resistance (HOMA-IR) (SMD = 1.217, p < 0.001). However, substantial heterogeneity across studies and potential publication bias were observed. Overall, GLP-1 RAs were associated with favorable improvements in key hormonal and metabolic markers compared with metformin in women with PCOS, including reductions in androgen levels and improvements in insulin resistance. Although the available evidence is limited by a small number of short-term trials, the findings are encouraging. Larger, well-designed randomized controlled studies with longer follow-up durations are needed to confirm the sustainability of these effects and to support their translation into routine clinical practice, with standardized reporting of adverse events and treatment discontinuation to better characterize safety and tolerability.

## Introduction and background

Worldwide, 6-20% of women in their reproductive years suffer from polycystic ovary syndrome (PCOS), a complex endocrine condition [1]. Ovulatory dysfunction, hyperandrogenism, and polycystic ovarian morphology are its defining characteristics. It is also frequently linked to metabolic disorders, including insulin resistance (IR), obesity, dyslipidemia, and an elevated risk of cardiovascular disease and type 2 diabetes mellitus (T2DM). One of the main causes of anovulatory infertility, PCOS presents serious long-term health hazards that require an efficient pharmaceutical approach to treat metabolic and reproductive issues [2].

Metformin, an insulin-sensitizing biguanide, has long been a cornerstone in PCOS treatment because it lowers hepatic gluconeogenesis, enhances insulin sensitivity, and facilitates weight loss. It has demonstrated efficacy in regulating menstrual cycles, lowering androgen levels, and improving ovulatory function. However, its clinical response is inconsistent, and its gastrointestinal side effects (e.g., nausea, diarrhea, stomach pain) frequently result in poor adherence and cessation [3].

Glucagon-like peptide-1 (GLP-1) receptor agonists (RAs) have recently shown promise as an alternative treatment for PCOS-related metabolic dysfunction. GLP-1 RAs, which were first created to treat type 2 diabetes, improve metabolic outcomes and cause considerable weight reduction by increasing glucose-dependent insulin secretion, suppressing glucagon release, slowing stomach emptying, and promoting satiety. Research indicates that GLP-1 RAs may be more effective than metformin in improving insulin sensitivity and helping people lose weight, but it is yet unknown how they may affect hormonal and reproductive outcomes like ovulation, hyperandrogenism, and regular menstruation [4,5].

While previous systematic reviews have explored this comparison, many have been limited in scope, relying on a narrow selection of databases or including non-randomized studies. Additionally, key aspects such as treatment adherence, long-term effects, and subgroup differences (e.g., lean vs. obese PCOS phenotypes, varying degrees of insulin resistance) remain underexplored. The purpose of this study is to evaluate the effects of GLP-1 RAs and metformin on metabolic, reproductive, and hormonal outcomes in individuals with PCOS.

## Review

Methods

The Preferred Reporting Items for Systematic Reviews and Meta-Analyses (PRISMA) criteria were followed in this systematic review and meta-analysis [6].

*Eligibility Criteria*

Study eligibility was assessed using predefined inclusion and exclusion criteria, as summarized in Table 1.

**Table 1:** Inclusion and exclusion criteria for study selection NIH: National Institutes of Health; GLP-1: glucagon-like peptide-1; PCOS: polycystic ovary syndrome

Inclusion Criteria	Exclusion Criteria
1. Study Design: Randomized controlled trials comparing GLP-1 receptor agonists with metformin in PCOS patients.	1. Reviews, meta-analyses, case reports, observational studies, and non-randomized research.
2. Population: Women diagnosed with PCOS based on the Rotterdam, NIH, or Androgen Excess Society criteria.	2. Studies with combination therapies (e.g., GLP-1 + metformin vs. placebo).
3. Intervention: Liraglutide, exenatide, and semaglutide are examples of GLP-1 receptor agonists.	3. Studies without original data or missing relevant outcome measures.
4. Comparator: Metformin monotherapy.	4. Animal or in vitro studies.

*Search Strategy*

To ensure a comprehensive evidence base, a systematic search was conducted across multiple electronic databases from database inception to April 2025. No filters were applied for age, population characteristics, or ethnicity. The databases searched included Google Scholar, PubMed, Web of Science, and Scopus. A combination of free-text keywords and Medical Subject Headings (MeSH) terms was used to identify relevant studies. Search terms covered PCOS, insulin resistance, hyperandrogenism, and metabolic syndrome, as well as pharmacologic agents such as metformin (N-dimethylbiguanide) and GLP-1 RAs (including liraglutide, exenatide, dulaglutide, and semaglutide). Keywords related to clinical outcomes, such as menstrual irregularities, ovulation, weight loss, and glucose metabolism, were also included. Boolean operators (“AND,” “OR”) were applied to refine the search strategy and retrieve the most relevant literature. In addition to the database search, reference lists of selected studies were manually screened to identify any additional eligible publications.

*Data Extraction*

To guarantee accuracy and consistency, two separate reviewers used a standardized data extraction form to obtain the data. Data was gathered on a number of important topics, such as participant characteristics (such as age and body mass index (BMI)) and study characteristics (such as author, year, country, study design, and sample size), intervention details (specifically the type of GLP-1 RA used, its dosage, and treatment duration), and details of the comparator group, including metformin dose and duration.

*Quality Assessment and Risk of Bias*

The Cochrane Risk of Bias tool version 2.0 (RoB 2.0) was used to evaluate the included studies' methodological quality [7] for RCTs. A number of bias types were assessed by this tool, including attrition bias (incomplete outcome data), performance bias (blinding of personnel and participants), detection bias (blinding of outcome assessment), selection bias (random sequence generation and allocation concealment), and reporting bias (selective reporting of outcomes). Additionally, the Grading of Recommendations, Assessment, Development, and Evaluations (GRADE) technique was used to evaluate the overall strength of the evidence.

*Statistical Analysis*

Comprehensive Meta-Analysis (CMA) version 3 software was used for all analyses [8], and a meta-analysis was carried out using a fixed-effects model to account for differences between trials. Effect sizes were computed according to the variable type. The 95% confidence intervals (CI) and the mean difference (MD) or standardized mean difference (SMD) were presented for continuous variables. Heterogeneity among studies was assessed using the I² statistic and Chi-square (Q test); low heterogeneity was indicated by values less than 25%, which supported the adoption of a fixed-effects model, moderate heterogeneity was indicated by results between 25% and 50%, which prompted sensitivity studies, and high heterogeneity was indicated by values over 50%. To assess publication bias, funnel plot analysis was performed for visual inspection. The asymmetry in the funnel diagram was statistically detected using Egger's regression test.

*Model Justification*

Because the included studies were very similar in terms of design, population characteristics, intervention type, comparator, and follow-up duration (all 12-week RCTs), a fixed-effect model was used in this meta-analysis. This model was chosen to estimate the average treatment effect under the presumption that sampling variation is the primary cause of observed differences and that the true effect is consistent across studies. In order to maintain comparability with earlier meta-analyses in this field and because the small number of included studies (n=4) limits the reliability and stability of random-effects variance estimates, the fixed-effect model was kept in place even though high heterogeneity was found in several outcomes. The fixed-effect model was deemed appropriate for summarizing the pooled effect in this review because of these factors.

Results

*Search Results*

**Figure 1:**
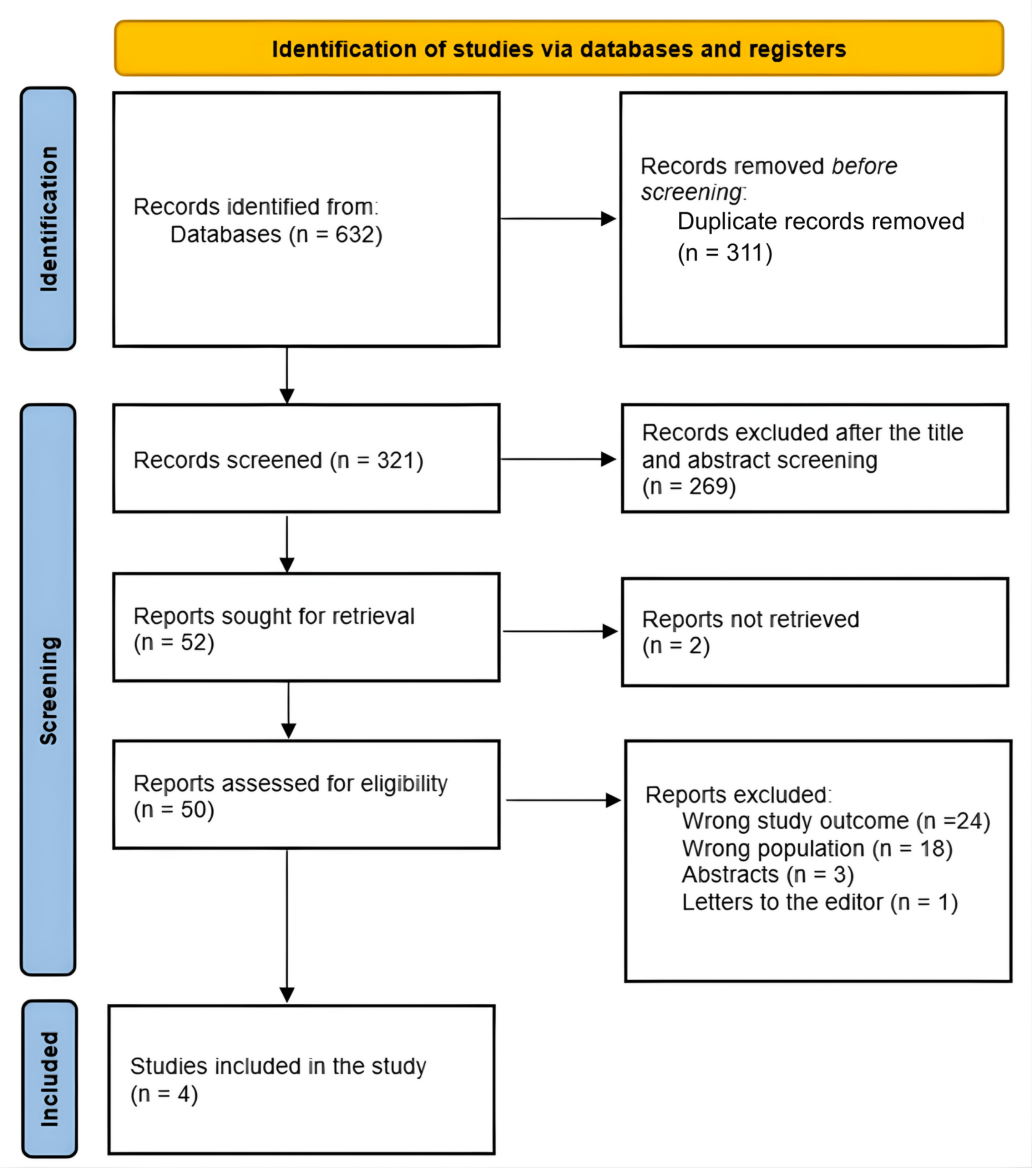
PRISMA flowchart showing study selection PRISMA: Preferred Reporting Items for Systematic Reviews and Meta-Analyses

A thorough search yielded 632 articles, from which 311 duplicates were removed. After analyzing the titles and abstracts of the remaining 321 papers, 269 were eliminated. Of the 52 full-text reports requested, only two could not be retrieved. Among the 50 full-text articles assessed for eligibility, 24 were excluded due to irrelevant study outcomes, 18 due to an unsuitable population, three were abstracts, and one was an editorial letter. Ultimately, four studies were included in the analysis after meeting the inclusion criteria. The study selection procedure is shown in Figure 1. 

*Characteristics of the Included Studies*

Table 2 demonstrates the sociodemographic characteristics of the studies taken into consideration. Four studies with a total of 218 women with PCOS (109 in the metformin group and 109 in the GLP-1 agonists) were included. All the studies were prospective RCTs [9-12]. Two studies were conducted in Slovenia [9,10], one in the United States [11], and one in China [12]. The studies by Jensterle et al. [9,10] compared metformin with liraglutide, while Tao et al. [11] and Zheng et al. [12] compared metformin with exenatide. All trials had a 12-week follow-up period, but differed in sample size and dosing strategies. Jensterle et al. [9,10] used smaller samples and focused on liraglutide titration, whereas Tao et al. [11] and Zheng et al. [12] used larger cohorts and evaluated fixed or flexible dosing of exenatide.

**Table 2:** Summary of the included studies MET: metformin; LIRA: liraglutide; EX: exenatide

Study (author, year)	Study design	Country	Age (years), mean ± years	Population	Follow-up (weeks)	Comparison	Dosage
Jensterle et al., (a) 2015 [9]	Prospective RCT	Slovenia	30.7 ± 7.9	27	12	MET versus LIRA	MET 1000 mg or LIRA 1.2 mg
Jensterle et al., (b) 2015 [10]	Prospective RCT	Slovenia	27.6 ± 7.2	28	12	MET versus LIRA	After a week, liraglutide was raised from 0.6 mg per day to 1.2 mg. Metformin began at 500 mg daily, gradually rising to 1000 mg twice daily.
Tao et al., 2021 [11]	Prospective RCT	United States	18-45	100	12	MET versus EX	MET was given 500–1000 mg twice a day, whereas EX was given 10–20 μg twice a day.
Zheng et al., 2019 [12]	Prospective RCT	China	18-40	63	12	MET versus EX	EX received a dose of 10 μg twice daily and MET at a dose of 1000 mg twice daily

*Meta-Analysis of Primary Effect Size*

**Figure 2:**
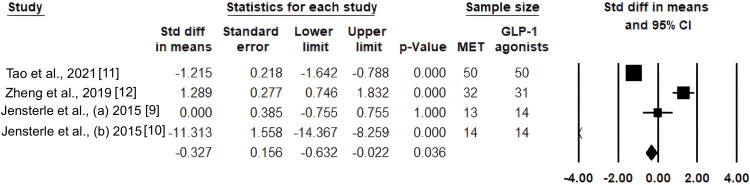
Forest plot of standardized mean differences in serum total testosterone between GLP-1 receptor agonists and metformin.

Results of total testosterone level in the GLP-1 RA and metformin groups: With a p-value of 0.036 and a pooled SMD of -0.327 with a 95% CI of -0.632 to -0.022, there is a statistically significant overall decrease in serum testosterone levels when using GLP-1 RAs as opposed to metformin (Figure 2).

**Figure 3:**
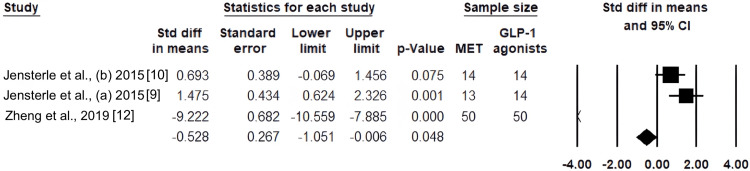
Standardized mean differences in DHEA-S between metformin and GLP-1 receptor agonists are shown in a forest plot. DHEA-S: dehydroepiandrosterone sulfate; GLP-1: glucagon-like peptide-1

Results of DHEA-S in the GLP-1 agonists and MET groups: Pooled SMD = -0.528, 95% CI: -1.051 to -0.006, p = 0.048, which indicates a statistically significant reduction in DHEA-S with GLP-1 RAs overall (Figure 3).

**Figure 4:**
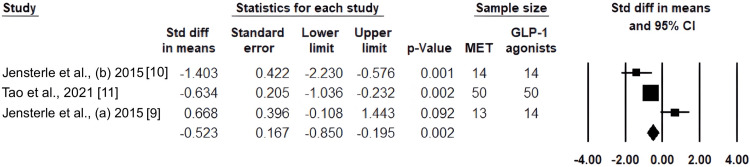
Standardized mean differences in androstenedione between metformin and GLP-1 receptor agonists are shown in a forest plot. GLP-1: glucagon-like peptide-1

Results of androstenedione in the GLP-1 agonists and MET groups: With a p-value of 0.002 and a pooled SMD of -0.523, the 95% CI ranged from -0.850 to -0.195. This suggests that GLP-1 RAs significantly lower androstenedione levels when compared to metformin (Figure 4).

**Figure 5:**
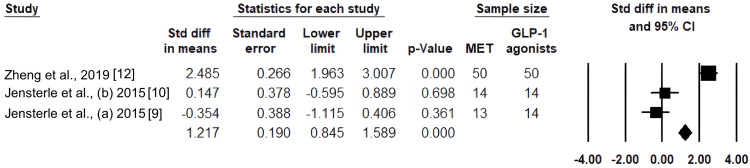
Standardized mean differences in HOMA-IR between metformin and GLP-1 receptor agonists are shown in a forest plot. HOMA-IR: homeostasis model assessment of insulin resistance; GLP-1: glucagon-like peptide-1

Results of HOMA-IR in the GLP-1 agonists and MET groups:* *With a 95% CI of 0.845 to 1.589 and a p-value of 0.000, the pooled SMD was 1.217, suggesting a statistically significant overall decrease in HOMA-IR in favor of GLP-1 RAs (Figure 5).

Publication bias

**Figure 6:**
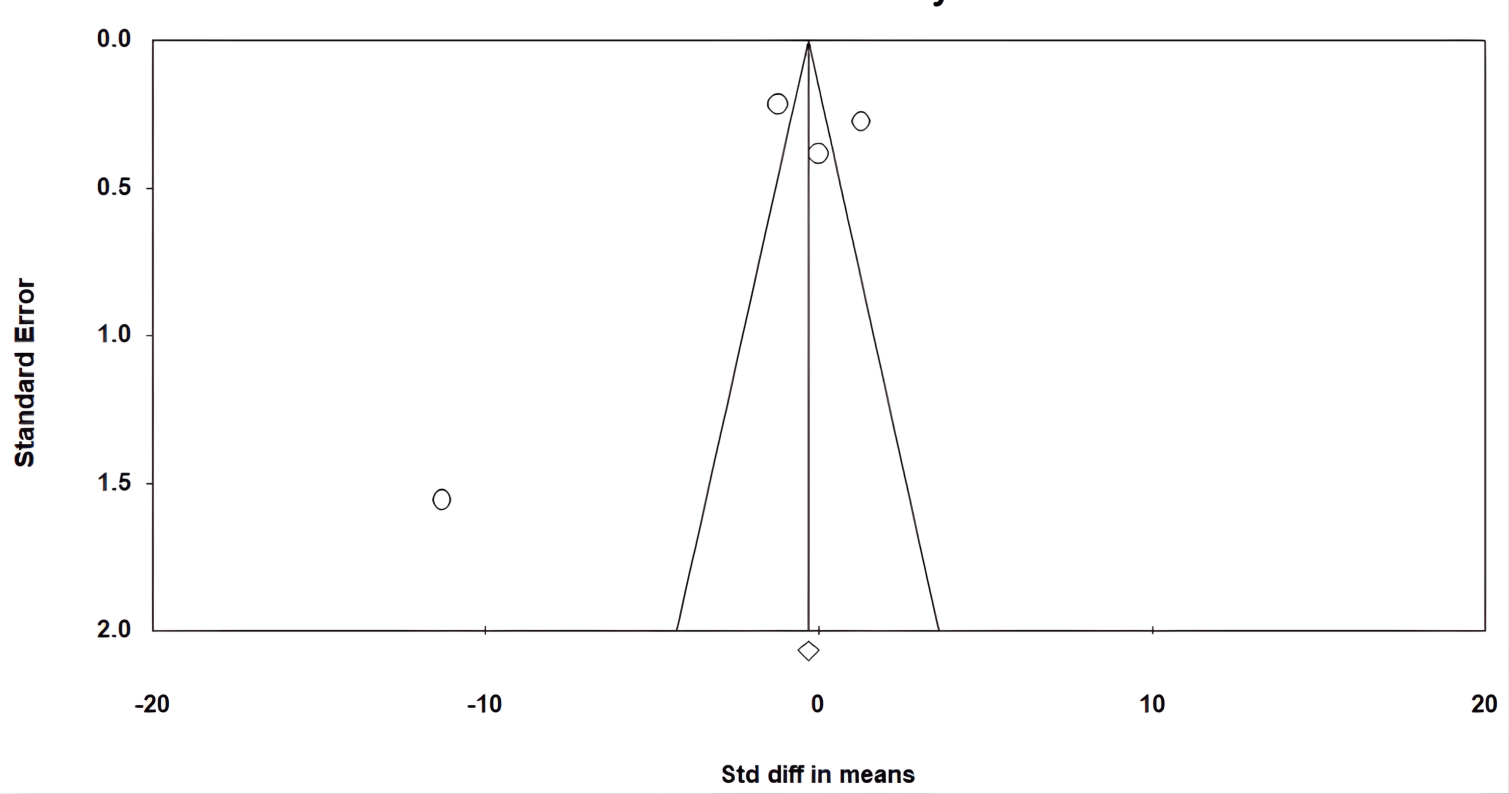
Funnel plot for publication bias.

The funnel plot demonstrated evident asymmetry, suggesting the presence of publication bias (I²=97.03%, P=0.000). This shows that the study's findings were rather strong (Figure 6).

Heterogeneity

Cochran's Q (Chi-square) test was used to assess heterogeneity for every result. All the outcomes that were part of the meta-analysis showed significant heterogeneity. Significant variation between studies was indicated by the exceptionally high heterogeneity of total testosterone (Q = 100, df = 3, I² = 97%). Both androstenedione (Q = 13.07, df = 2, I² = 85%) and DHEA-S (Q = 32.73, df = 2, I² = 94%) showed significant heterogeneity. With extremely high heterogeneity (Q = 46.4, df = 2, I² = 96%), HOMA-IR displayed the most inconsistent results.

*Risk of Bias Assessment*

**Figure 7:**
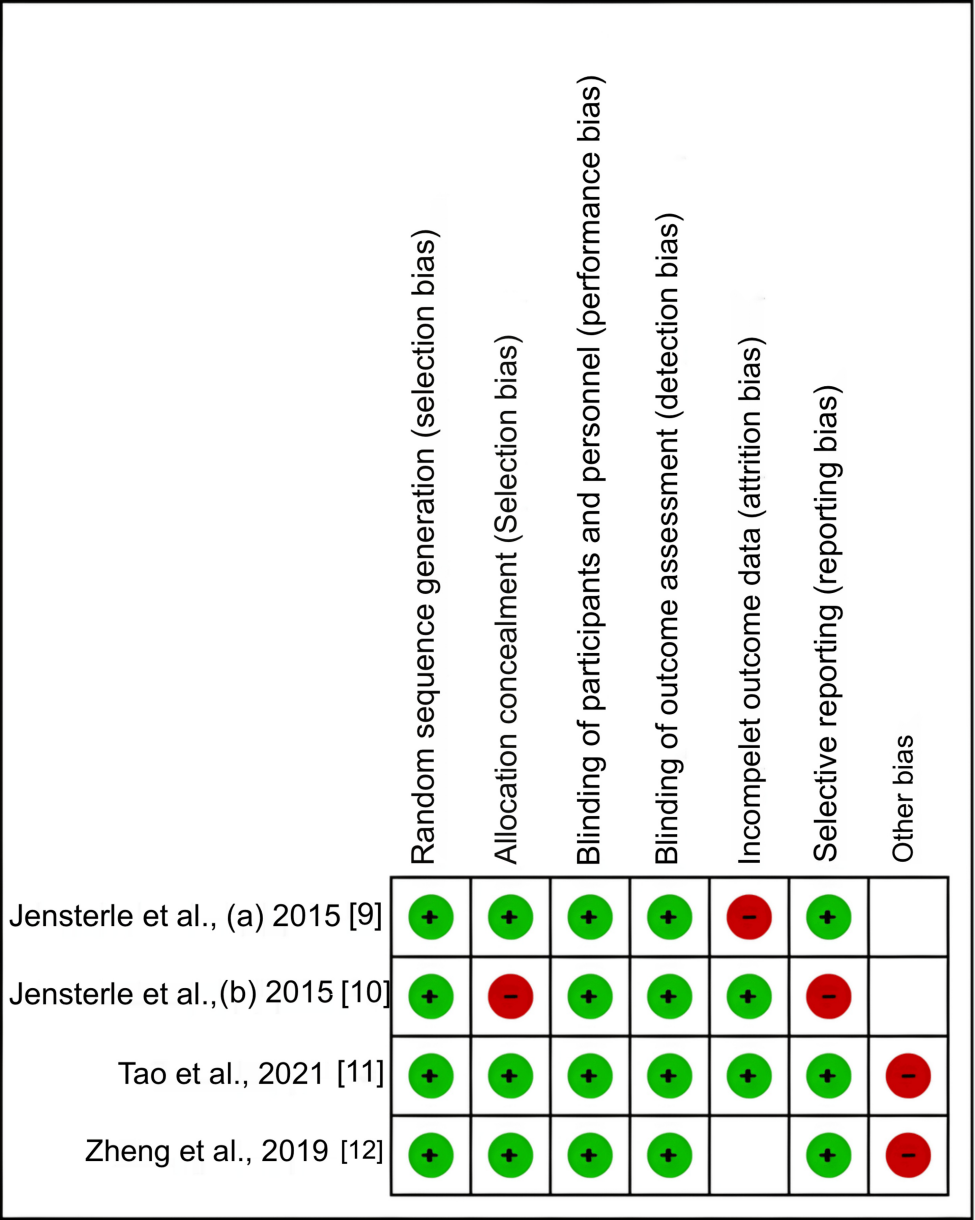
Cochrane risk of bias assessment.

Overall, the included studies demonstrated a generally low risk of bias across several domains, particularly in random sequence generation, allocation concealment, and blinding. Nevertheless, some concerns were observed in selective reporting and other sources of bias in individual studies (Figure 7).

Discussion

The purpose of this meta-analysis was to assess the reproductive, hormonal, and metabolic effects of GLP-1 RAs compared with metformin in women with PCOS. The results showed that in this demographic, GLP-1 agonists were more effective than MET.

The pooled analysis in this study showed a significant reduction in serum testosterone levels with GLP-1 RAs compared to metformin (SMD = -0.327, p = 0.036). The exact mechanism by which GLP-1 RAs affect androgen levels remains uncertain. Exenatide, however, has been shown in animal research to reduce blood testosterone levels and lessen hyperandrogenism in PCOS models [13]. Additionally, a systematic review by Niafar et al. found that serum testosterone was the only parameter to show a significant decrease after three months of GLP-1 RA treatment [14].

Exenatide improved granulosa cell layers and decreased the amount of cystic follicles, among other aspects of follicle shape, according to a study that used a DHEA-induced rat model of PCOS [13]. Our pooled analysis showed a significant reduction in DHEA-S levels with GLP-1 RAs compared to metformin (SMD = -0.528, p = 0.048). This section will examine the complex link between DHEA-S and insulin. According to certain research, there is a negative link between HOMA-IR and DHEA-S [15]. Others, however, have not discovered any meaningful correlation [16]. Perhaps a favorable association between insulin resistance and increased levels of adrenal precursor androgen. It is important to note that, in comparison to the gold standard diagnostic techniques, HOMA-IR is an inaccurate assessment and may not be able to detect insulin resistance in women with PCOS [17]. Ethnic distinctions could also be important; one study found that DHEA-S and HOMA-IR were negatively correlated in White patients but not in Black individuals [16]. Nevertheless, the study's Black cohort's small sample size limits the capacity to make definitive inferences.

Our analysis also showed a significant improvement in HOMA-IR with GLP-1 RAs compared to metformin (SMD = 1.217, 95%CI: 0.845-1.589, p < 0.001), indicating greater insulin sensitivity. Bo et al. reported that combination therapy with GLP-1 RAs reduced HOMA-IR (MD= -1.29; 95%CI= -2.38 to -0.21) compared to placebo [18]. In contrast, De Hollanda et al. reported no significant effect of GLP-1 agonists on HOMA-IR levels [19].

Additionally, we discovered that GLP-1 RAs significantly decreased androstenedione levels as compared to metformin (SMD = -0.523, p = 0.002). Likewise, Ye et al. found that exenatide had a greater impact on sex hormones such as androstenedione than metformin [20]. Also, Yang et al. found that among the treatments compared for reducing androstenedione levels, GLP-1 RAs were the most effective, with an MD of -3.06 (95%CI: -5.53 to -0.62). This was followed closely by the combination therapy GLP-1 RAs + metformin (MD = -2.97) [21].

The results of this meta-analysis and comprehensive review provide valuable clinical information on PCOS treatment. Originally created to treat type 2 diabetes, GLP-1 RAs have shown better results than metformin in lowering blood levels of testosterone, DHEA-S, androstenedione, and HOMA-IR, among other important clinical indicators in women with PCOS. These improvements suggest not only better metabolic outcomes but also potential reproductive benefits. Given the challenges many patients face with metformin’s side effects and inconsistent efficacy, GLP-1 RAs may serve as a valuable alternative, particularly for patients struggling with obesity or insulin resistance. However, given their higher cost and potential side effects such as gastrointestinal discomfort, individualized treatment plans remain crucial. Clinicians should consider patient phenotype, metabolic status, and treatment tolerance when choosing between or combining therapies.

*Clinical Implications*

The findings of this meta-analysis highlight important clinical implications for the management of PCOS. GLP-1 RAs demonstrated superior effects on insulin resistance and androgen reduction compared with metformin, suggesting they may be preferable for patients with significant metabolic dysfunction or hyperandrogenism. Given metformin’s frequent gastrointestinal side effects and high discontinuation rates, GLP-1 RAs represent a viable alternative for patients who do not tolerate or respond adequately to metformin. Furthermore, the improvements in androgen levels may translate into better ovulatory and reproductive outcomes, although long-term trials evaluating menstrual regularity, ovulation rates, and fertility outcomes are still lacking. Clinicians should consider patient-specific factors, including obesity, metabolic risk, and treatment adherence, when selecting between GLP-1 RAs and metformin.

*Strengths and Limitations*

This study has several notable strengths. The exclusive inclusion of RCTs enhances the methodological rigor and strengthens the validity of the pooled findings. Adherence to PRISMA guidelines, use of comprehensive database searches, and application of established assessment tools, including the Cochrane Risk of Bias Tool and GRADE framework, further improve the reliability of the evidence. By focusing on direct comparisons between GLP-1 RAs and metformin, the analysis provides clearer insights into their relative efficacy. Additionally, evaluating both metabolic and hormonal outcomes offers a broad and clinically relevant perspective on therapeutic effects in PCOS.

However, important limitations must be acknowledged. The small number of included trials (n = 4) and modest total sample size reduce statistical precision and limit the generalizability of the results. Clinical heterogeneity-arising from variations in drug type, dosage schemes, population characteristics, and baseline metabolic severity-likely contributed to the high I² values observed. The short 12-week duration of all studies restricts conclusions regarding long-term metabolic improvements, fertility outcomes, or sustained hormonal changes. Moreover, asymmetry in the funnel plot suggests potential publication bias, raising the possibility that positive findings may be overrepresented. Finally, variability in diagnostic criteria and hormonal assessment methods across studies may account for some inconsistency in treatment effects.

*Future Directions*

Future research should include large, multicenter RCTs with longer follow-up to evaluate the sustained metabolic, hormonal, and reproductive effects of GLP-1 RAs. Priority areas include ovulation, menstrual regulation, fertility outcomes, and long-term cardiometabolic risk. Comparative trials between different GLP-1 RAs are needed to identify drug-specific benefits, alongside subgroup analyses based on obesity, insulin resistance, and PCOS phenotype. Investigating combination therapies with lifestyle interventions or metformin may also clarify potential additive effects. Finally, standardized diagnostic criteria and hormonal assays are essential to improve consistency across future studies.

## Conclusions

In women with PCOS, GLP-1 RAs may offer advantages over metformin in reducing androgen levels and improving insulin resistance. These findings support a potential role for GLP-1 receptor agonists as an adjunct or alternative to metformin, particularly in individuals with obesity and insulin resistance. Nevertheless, the evidence is limited by the short study duration, small sample sizes, and possible publication bias. Further large, long-term RCTs are warranted to evaluate sustained clinical benefit, prioritize patient-centered outcomes, and clarify subgroup responses, alongside standardized adverse-event reporting and documentation of treatment discontinuation to better define tolerability and safety and guide individualized treatment strategies in PCOS.
